# Immune system correlates of extensive limb swelling in response to conjugated pneumococcal vaccination

**DOI:** 10.1038/s41541-018-0059-3

**Published:** 2018-05-18

**Authors:** Mike Recher, Julia R. Hirsiger, Marc B. Bigler, Martin Iff, Barbara Lemaître, Kathrin Scherer, Peter Häusermann, Claire-Anne Siegrist, Christoph T. Berger

**Affiliations:** 1 Immunodeficiency Clinic and Laboratory, Medical Outpatient Unit, Department Internal Medicine and Department Biomedicine, University Basel Hospital, Basel, Switzerland; 2 Translational Immunology, Department of Biomedicine, University Basel Hospital, Basel, Switzerland; 3 Internal Medicine Praxis, Reinach, Switzerland; 4 Center for Vaccinology, Geneva University Hospitals, Geneva, Switzerland; 5 Allergy Unit, Department of Dermatology, University Basel Hospital, Basel, Switzerland; 6 Department of Dermatology, University Basel Hospital, Basel, Switzerland; 7 Clinical Immunology and Vaccination Clinic, Medical Outpatient Clinic, Department of Internal Medicine, University Basel Hospital, Basel, Switzerland

## Abstract

Pneumococcal conjugate vaccine (PCV) is recommended for adults with chronic disease. Extensive limb swelling (ELS) is an acute vigorous local inflammatory reaction following vaccination. Predisposing immune system correlates and the influence of ELS on vaccine responses are not known. Here, we report a case of a 67-year-old woman with a history of multiple pneumonias who had a detailed immunological work-up pre-vaccination because of suspected immunodeficiency. Four days following a first vaccination with PCV13 she developed ELS—mimicking erysipelas. Treatment with 20 mg cortisone completely alleviated symptoms within 2 days. Skin biopsy showed a dense dermal and subdermal infiltration dominated by CD4^+^ T cells and macrophages. In a multiplexed serotype-specific measurement of the anti-pneumococcal IgG response, the patient showed very broad and strong vaccine responses. Pre-vaccination titers were low for the vaccine serotypes. We did not find in vivo nor in vitro evidence of an excessive T cell response to the diphtheria-derived PCV carrier protein. However, we could demonstrate a high antibody titer to a non-vaccine serotype, indicating in vivo pre-exposure to pneumococcus bacteria. Thus, traces of pneumococcal proteins included in PCV13 may have boosted pre-existing pneumococcus-specific T helper cells, which subsequently orchestrated ELS. Our case raises awareness for the risk of vaccine-induced ELS, especially in patients with a history of recurrent pneumococcal disease and thus partial immunity.

## Introduction

Invasive pneumococcal infection has a high morbidity and mortality,^1^ often preventable by a single vaccination with the pneumococcal conjugate vaccine (PCV).^2^ The Capita trial indicating vaccine-conferred protection from community-acquired pneumonia (CAP) in immunocompetent elderly extended the use of PCV beyond patients with chronic disease.^2,3^ The 13-valent conjugate pneumococcal vaccine (PCV13) contains the capsular polysaccharides (PS) of 13 serotypes at 25 μg each. These are covalently bound (conjugated) to a diphtheria toxoid carrier protein CRM_197_. This carrier protein is processed by dendritic cells and PS-specific B cell and presented to CD4^+^ T helper cells.^4^ Upon activation these provide activation signals to the PS-specific B cells thereby enhancing antibody class switch and memory formation. Extensive inflammatory skin reactions at the vaccination site, also known as extensive limb swelling (ELS),^5^ have been described following PCV^6^ and pneumococcal polysaccharide vaccine (PPV).^7–10^ However, since pre-existing immunity at the time of vaccination is usually unknown, there are no immune system correlates known to predispose to such reactions. In addition, it is not clear whether such reactions and/or the anti-inflammatory treatment they require are associated with strong or impaired humoral vaccine responses.

## Results

**Fig. 1:**
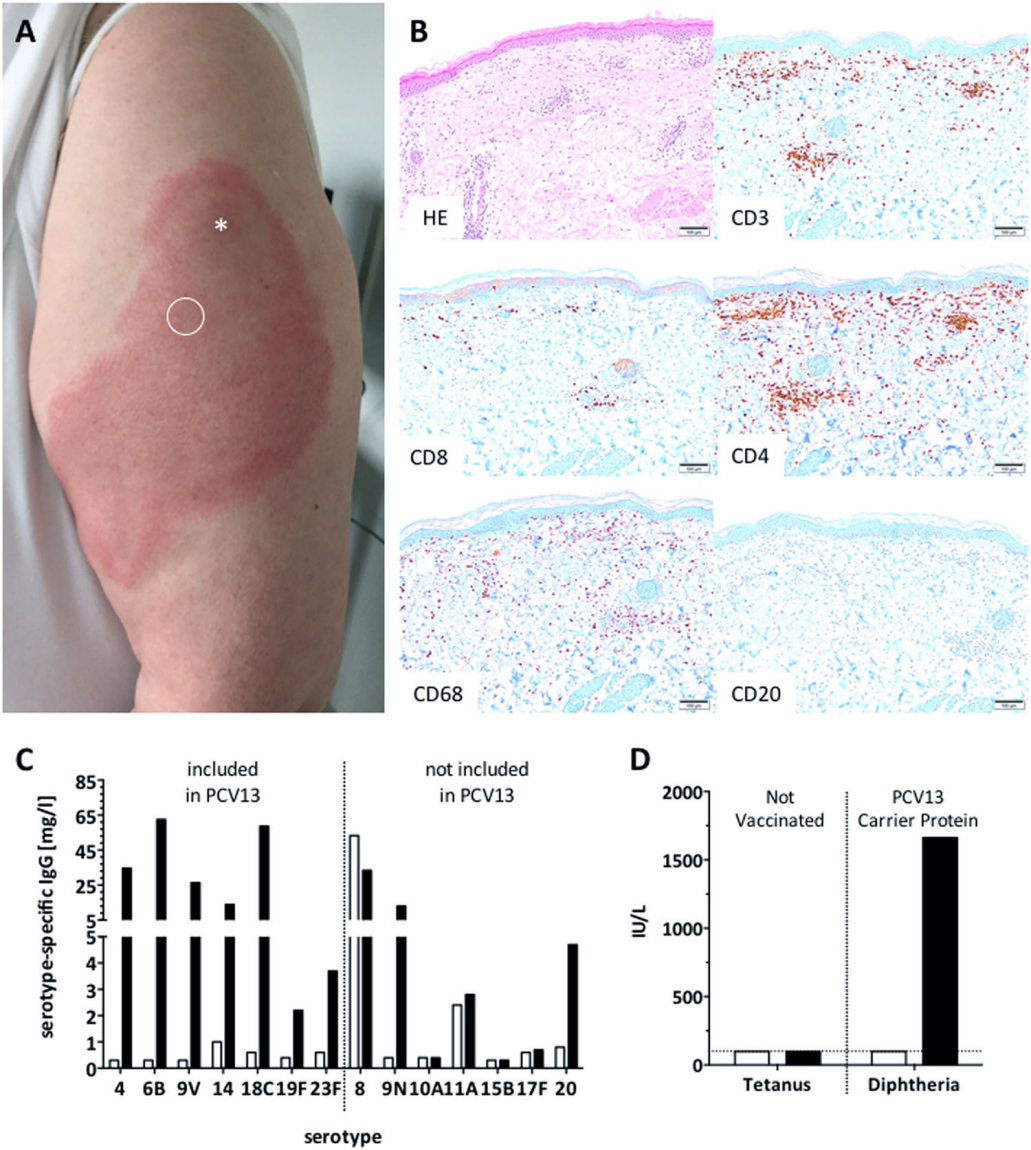
Strong local injection site reaction 4 days following PCV13 vaccination. **a** Circle indicates biopsy site, asterisk indicates the vaccine injection was done. **b** Skin biopsy shows infiltration of mononuclear cells, mostly consisting of CD4^+^ T cells and CD68^+^ macrophages, while B cells were virtually absent. Scale bar indicates 100 μm. **c** Serotype-specific IgG responses pre-vaccination (open bars) and 4 weeks post-vaccination (black bars) toward vaccine and non-vaccine strains. Values below lower detection limit (<0.3 mg/L) were set to 0.3 mg/L. **d** Pre-PCV vaccination titers against tetanus and diphtheria were low/undetectable. Post-PCV vaccination diphtheria titers (i.e., anti-carrier protein) were high

Here we report an extensive inflammatory skin reaction that occurred at the site of vaccination in a 67-year-old Caucasian woman following a single dose of PCV13 (Fig. 1). Prior to PCV vaccination she underwent a detailed immunological work-up at our immunodeficiency clinic because of recurrent lower respiratory tract infections. Over the past 6 years, she experienced three CAP, once with sepsis. Bacterial blood cultures and search of urinary pneumococcal and legionella antigens were performed after initiation of antibiotic therapy. No causative agent could be isolated for any of the three episodes. Patient history was remarkable for two episodes of diverticulitis, recurrent herpes labialis (“cold sore”; reactivation rate <1/year^11^ and not for several months preceding PCV administration), obesity (BMI 39 kg/m^2^), and non-insulin-dependent diabetes mellitus. No opportunistic infections were documented. She was not taking any immunosuppressive drugs. She had been vaccinated according to the Swiss vaccination guidelines.^12^ The last tetanus/diphtheria booster dose had been administered 17 years ago. She had never received any pneumococcal vaccine. The physical examination and CT scan imaging were normal, with no evidence for chronic pulmonary disease or lympho proliferation. Body pletysmography was normal, excluding asthma or chronic obstructive lung disease. Immunologic analysis in the clinical routine showed normal total serum IgG, IgM, IgA, and IgG_1–4_ subclass levels. Lymphocyte (T vs. B vs. NK cell) and B cell subset distribution (naive vs. memory vs. marginal zone-like B cells) were normal (Table 1). Functional complement screening revealed normal classical and alternative pathways (Table 1). In summary, there was no evidence of an underlying immunodeficiency that would have predisposed to the recurrent infections. Diabetes and obesity were potential risk factors.

**Table 1:** Basic immunological work-up (pre-PCV vaccination)

		Dimension	Norm
IgG	7.4	g/L	7.0–16.0
IgG1	5.6	g/L	4.9–11.4
IgG2	1.79	g/L	1.5–6.4
IgG3	0.56	g/L	0.2–1.1
IgG4	0.13	g/L	0.08–1.4
IgM	0.92	g/L	0.4–2.3
IgA	0.97	g/L	0.7–4.0
CD4^+^ T cells	1329	/μl	404–1612
CD8^+^ T cells	602	/μl	220–1129
Natural killer cells	231	/μl	84–724
B cells	239	/μl	80–616
Naive B cells (CD19^+^CD27^−^IgD^+^IgM^+^)	60.4	%	25.1–92.4
Marginal zone-like B cells (CD19^+^CD27^+^IgD^+^IgM^+^)	24.1	%	3.1–59.7
Class switched memory B cells (CD19^+^CD27^+^IgD^−^IgM^−^)	9	%	2.4–32.6
CD21low B cells (CD19^+^CD21^−^CD38^−^)	1.9	%	0.5–4.7
Transitional B cells (CD19^+^CD38^+^IgD^−^IgM^+^)	2.1	%	0.3–2.9
Plasma blasts (CD19^+^CD38^++^IgD^−^IgM^−^)	0.2	%	0.1–3.0
Classical complement activation	112	%	69–129
Alternative complement activation	109	%	30–113
Tetanus toxoid IgG	<100	IU/L	>100
Diphteria toxoid IgG	<100	IU/L	>100

Given the history of recurrent lower respiratory tract infections, the patient was vaccinated with PCV13 (Prevenar13, Pfizer). Three days later, she developed a progressive, extensive, and painful swelling and erythema of about 10 × 20 cm (4 × 8 inches), affecting almost all of her upper arm around the site of injection (Fig. 1a). Given the strong pain and extensive local inflammatory reaction, she received glucocorticosteroid (20 mg prednisone daily) and an antihistaminic for 4 days, with a favorable evolution. A skin biopsy obtained on day 4 following vaccination, and 46 h following the first dose of prednisone/antihistamine, showed a dense dermal and subdermal perivascular-intensified infiltration dominated by CD4^+^ T cells and CD68^+^ macrophages. This pathology is classical of a type IV delayed hypersensitivity reaction—as seen with a positive Mantoux tuberculosis skin test^13^ (Fig. 1b). CD8^+^ T cells were very sparse and B cells were almost completely absent (Fig. 1b). Pre-vaccine and 4 weeks post-vaccine anti-pneumococcal IgG levels were determined in a multiplexed serotype-specific assay testing a total of 14 serotypes: 7 that are included in PCV13, and 7 that are not (Fig. 1c). Seroresponses were very vigorous against all tested PCV13 serotypes plus serotypes 9N and 20, not included in PCV13. Strikingly, a very high pre-vaccination IgG titer was observed against the non-PCV13 serotype 8, suggesting prior infection with this serotype (Fig. 1c). There was a humoral response against the diphtheria carrier protein CRM_197_, as anti-diphtheria titers increased following PCV13 vaccination, which was not observed for an irrelevant control protein (tetanus) (Fig. 1d). Notably, this anti-diphteria titer increase was recorded before the subsequent DiTe booster dose that was given 4 weeks after the PCV vaccination.

In vitro B cell activation 2 months after PCV13 vaccination showed a strong PCV13-induced B cell proliferation and plasmablast induction, and also a high spontaneous B cell proliferation rate in the absence of exogenous antigen (Supplementary Fig. S1). In vitro T cell responses (2 months post PCV13 vaccination and 1 month post DiTe booster vaccination), as measured by directly ex vivo ELISpot and T cell proliferation assay following in vitro stimulation with DiTe and PCV vaccines were measurable, but not different from control individuals (Supplementary Fig. S2).

## Discussion

Several immune-mediated adverse reactions may be induced by immunization. Neither the clinical nor the biological observations are suggestive of a type I (immediate IgE-mediated) or type II (antibody-dependent cytotoxic) reaction to the vaccine. A type III immune complex-mediated reaction is unlikely as it typically occurs within 12 h after vaccination, and the patient did not have high pre-existing antibodies against any of the vaccine antigens assessed (Fig. 1c).^14,15^ In addition, we did not find histological evidence of immune complex deposition, although their detection might have been prohibited due to formalin fixation (data not shown). In patients with Behçet’s disease, an auto-inflammatory syndrome, severe early-onset local reactions have been reported following PPV, possibly due to hyperactive innate immunity.^16^ In our case, in vitro stimulation of patient-derived peripheral blood mononuclear cells (PBMC) with lipopolysaccharide (LPS) resulted in secretion of the innate cytokines (TNF-α and IL-6) comparable to levels induced in PBMC of control individuals (Supplementary Fig. S3). In addition, auto-inflammatory reactions usually start early (<24 h) post-vaccination. This argues against innate immune system over-reactivity driving ELS in our patient.^17^

In contrast, the clinical pattern of a delayed (≥48 h) and progressive local reaction is evocative of type IV delayed type hypersensitivity. This reaction is mediated by strong recall responses of memory CD4^+^ T helper cells and secondary macrophage activation—as seen in response to tuberculin skin injection. A plausible source of memory T cell stimulating proteins/peptides was the diphtheria-related carrier protein CRM_197_ in PCV. This, however, appears very unlikely since (1) baseline anti-diphtheria titers were below the protective range; (2) the patient tolerated a DiTe booster vaccination (4 weeks after the ELS) without any injection site reaction (ISR); and (3) in vitro T cell assays indicated no augmented responsiveness to diphtheria toxoid (Supplementary Fig. S2).

Having ruled out anti-carrier protein T cell reactivity, the most likely explanation would be a memory T helper cell response against pneumococcal proteins included in PCV13.^18^ This is supported by the high pre-existing anti-serotype 8 response and the vaccine-induced responses to non-PCV13 serotypes. Those T cells might have been recruited from the central T cell memory pool of the draining lymph nodes. Following PCV13 vaccination, such memory T cells might have been activated to drive ELS, in concert with (CD4^+^ T cell-)activated macrophages. Patch skin testing performed with DiTe, PPV23, and PCV13 several months later induced no skin inflammation. The sensitivity of skin tests for such reactions is, however, known as low.^19^

Data on the frequency of ELS following PCV are sparse. However, in a detailed assessment of ELS reported to the vaccine adverse event reporting system (VAERS), PPV was the most frequent vaccination associated with ELS in older patients.^10^ In this largest assessment of ELS associated with vaccination, the authors compiled all cases reported to the (VAERS) between 1990 and 2003. Each year the VAERS receives >14,000 possible vaccine-related adverse events. Seventy-nine of 497 identified ELS cases occurred in subjects >65 years, and 70.5% of those were associated with PPV vaccination. Since these are data from a reporting system, and given the overall frequency of local ISRs of any severity, these studies preclude mechanistic insights.^7^ Reports of small case series, however, also suggested an association of ELS following PPV vaccination and previous exposure to PPV or pneumococci.^8,9^

In summary, we report ELS occurring 4 days following PCV vaccination in a patient with a history of repeated CAP. In-depth immune system phenotyping suggests that pneumococcal-specific memory T helper cells might be responsible for the strong local reaction in the setting of a strong infection-induced immunity. Despite early systemic glucocorticosteroid administration, the 4-week post-vaccination titers were very high. With the expected increased usage of PCV in adults,^2^ clinicians need to be aware of vaccination-induced ESL, especially in those with previous (invasive) pneumococcal disease.

## Methods

The patient gave written informed consent and the study was conducted in accordance with the ethics committee of northwestern Switzerland. All patient information has been anonymized. Pre-vaccine and 4 weeks post-vaccine anti-pneumococcal IgG levels were determined in a multiplexed, electrochemiluminescence-based serotype-specific assay (Mesoscale Discovery^20,21^). Immunohistochemistry was performed using an automated slide stainer (BenchMark Ultra, Ventana Medical Systems) using the Optiview DAB IHC Detection Kit (Venata Medical Systems). The antibodies used were directed against CD3 (clone 2GV6, Ventana), CD4 (clone SP35, Ventana), CD8 (clone SP57, Ventana), CD20 (clone L26, Ventana), or CD68 (clone PG-M1, Dako). B cell proliferation of cell trace violet (CTV)-labeled PBMC (CTV Proliferation Kit, Thermofisher Scientific, Switzerland) was assessed after 6 days stimulation with PPV (Pneumovax23, Merck), PCV13 (Prevenar 13, Pfizer), or DiTe (dT-pur, Berna) vaccines in a 1:100 dilution. Cells cultured in media alone served as negative control, OKT3/aCD28 (100 ng/mL each) and CD40L (100 ng/mL) and IL21 (100 ng/mL)-activated PBMC served as positive control for T, resp. B cells. Data was acquired on a multicolor flow cytometer (BD LSRFortessa). B cells were gated on CD3^−^CD19^+^ and T cells on CD3^+^CD4^+^ or CD3^+^CD4^−^ single, live lymphocytes. Background-corrected (Proliferation_Stimulus_/Proliferation_Media_) frequencies of proliferated according to manufacturer’s instructions. To assess plasmablast generation after in vitro stimulation with the vaccines, PBMC were stimulated as described above and cells were cultured for 5 days. B cells were defined as CD3^−^CD14^−^CD16^−^CD19^+^ single, live lymphocytes and plasma blasts defined as the CD38^high^ CD27^high^ subset. Directly ex vivo T cell IFNγ-ELISpot was performed by incubating fresh PBMC for 48 h in the presence of the different vaccines using 100,000 PBMC per well.^22^ Spot-forming cells per million PBMC was calculated. To test for an over active innate immunity 1 million PBMC were stimulated with 50 ng/mL LPS (TLR4 ligand) and cultured overnight. Culture supernatants were analyzed by ELISA for Interleukin-6 (Peprotech, Switzerland IL-6-ELISA: 900-M16) and TNF-α (Peprotech, Switzerland TNFα-ELISA: 900-TM25).

### Data availability

All data generated or analyzed during this study are included in this published article (and its Supplementary Information files).

## Electronic supplementary material

Supplementary Figures S1-S3
