# Supplementary material for: Immune system correlates of extensive limb swelling in response to conjugated pneumococcal vaccination
Source: NPJ Vaccines. 2018 May 18;3:17. doi: 10.1038/s41541-018-0059-3 (PMC5959910; doi:10.1038/s41541-018-0059-3)
Supplement: Supplementary file 1 — Supplementary Figures S1-S3 [file 41541_2018_59_MOESM1_ESM.pdf]

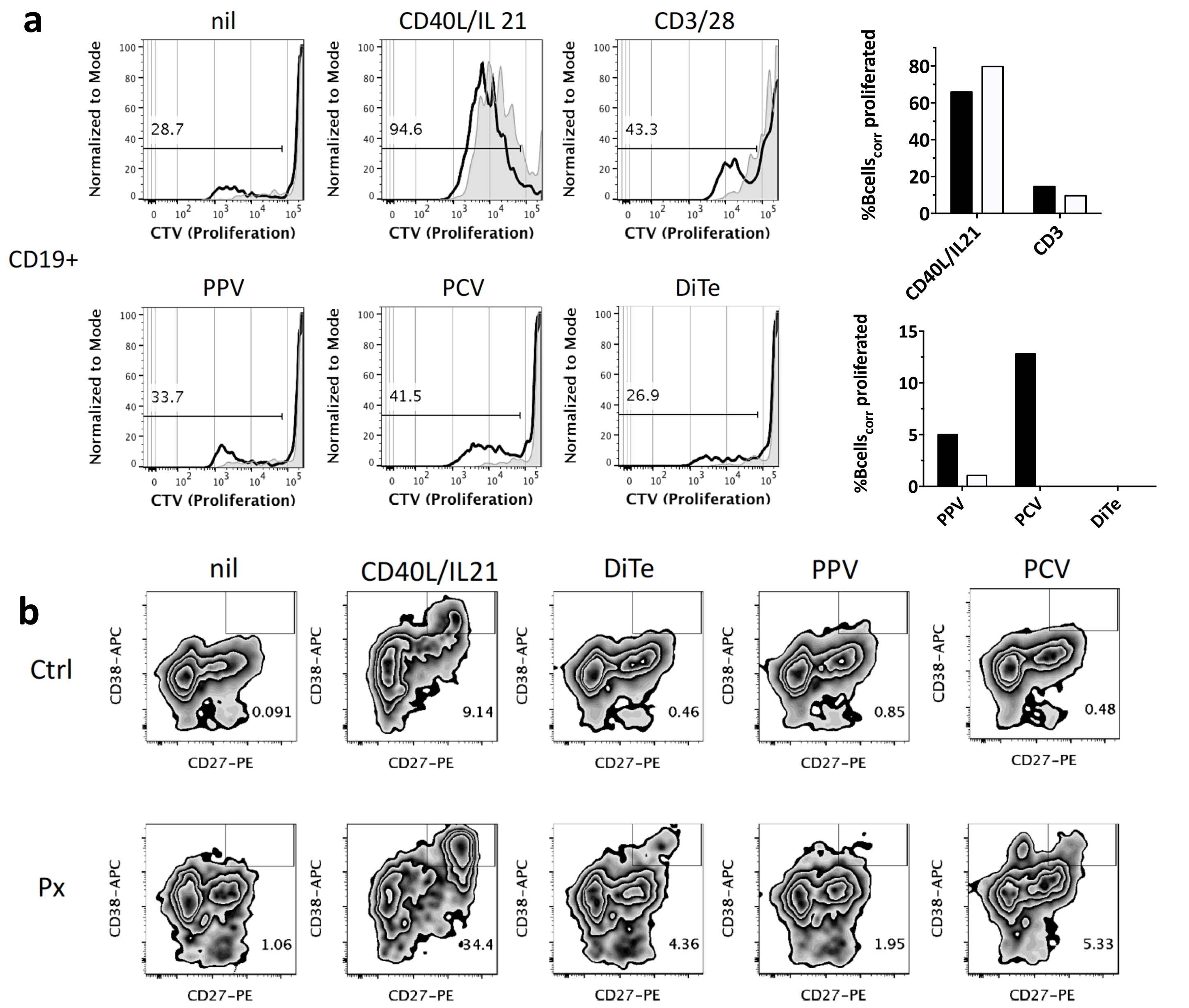

Supplementary Figure S1: Characterization of the B cell response. (a) PBMC of the patient two months after PCV and one month after DiTe vaccination were activated using different stimuli and proliferation was monitored after six days using the cell trace violet (CTV) proliferation kit (Thermofisher Scientific, Switzerland) according to manufacturer's instructions. Cells were stimulated with PPV (Pneumovax23, Merck), PCV13 (Prevenar 13, Pfizer) or DiTe (dT-pur, Berna) vaccines in a 1:100 dilution. Cells cultured in media alone (nil) served as negative control, OKT3/aCD28 (100 ng/mL each) and CD40L (100 ng/ml) and IL21 (100 ng/ml) served as positive control for T, resp. B cells. Data was acquired on a multicolor flow cytometer (BD LSRFortessa). Cells were gated on CD3-CD19+ single, live lymphocytes. Background-corrected ($Proliferation_{Stimulus}/Proliferation_{media}$) frequencies of proliferated B cells are shown (black lines in histograms and black bars), and compared to an age- and gender matched control (grey shaded area in the histograms, and open bars). Overall B cell proliferation capacity was comparable, but the patient showed a higher proliferation in response to both pneumococcal vaccines. Notably, the control was not PCV- or PPV-vaccinated (b) PBMC of the patient two months after PCV-vaccination (lower panels) and an unvaccinated control (upper panel) were stimulated as described above and cells were cultured for 5 days. B cells were defined as CD3-CD14-CD16-CD19+ single, live lymphocytes and plasma blasts defined as the CD38high CD27high subset. The patient showed a robust plasma blast expansion towards DiTe, PPV and PCV and an overall strong plasma blast induction by the positive control.

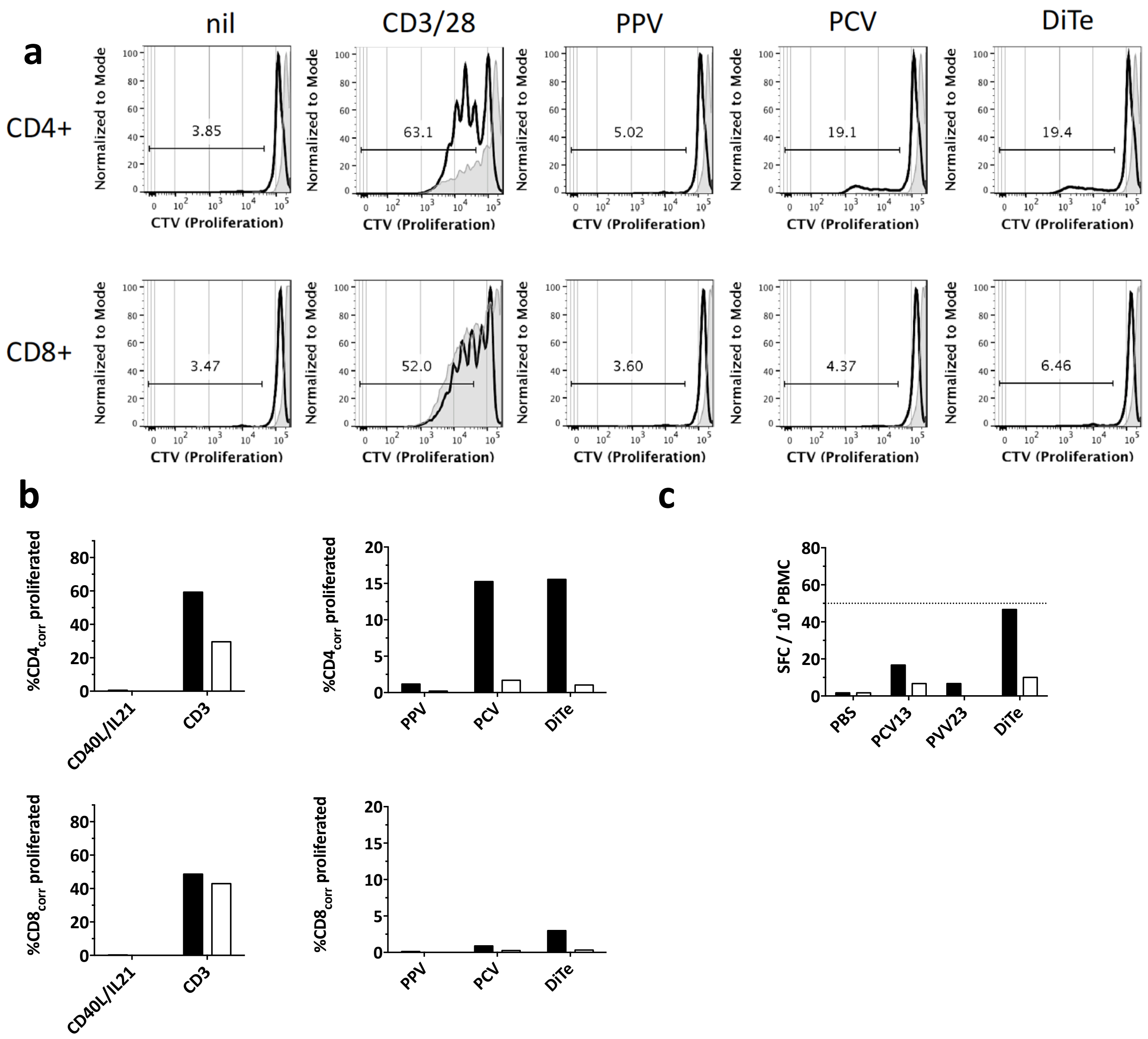

Supplementary Figure S2: Characterization of the T cell responses. (a) PBMC of the experiment described in Supplementary Figure S1 gated on CD3+CD4+ (upper panel) and CD3+CD4- (='CD8', lower panel) Tcells. (b) Proliferation (Thermo Fisher CTV Proliferation kit: C34557) was compared as background- corrected ($Proliferation_{Stimulus}$/$Proliferation_{media}$) frequencies of proliferated T cells (patient = black lines in histograms and black bars; control = grey shaded area in the histograms, and open bars). The patient showed stronger proliferation of the CD4 T cells upon OKT3 as well as against the two protein-containing vaccines: PCV and Tetanus. Samples were tested two months post-PCV and one month post DiTe. (c) Directly ex vivo IFNg-ELISpot. Fresh PBMC cells were incubated for 48h in the presence of the different vaccines (see S1) using 100'000 PBMC per well. Spot forming cells (SFC) per million PBMC was calculated. The patient showed an increased DiTe response after the DiTe vaccination, but overall responses were weak.

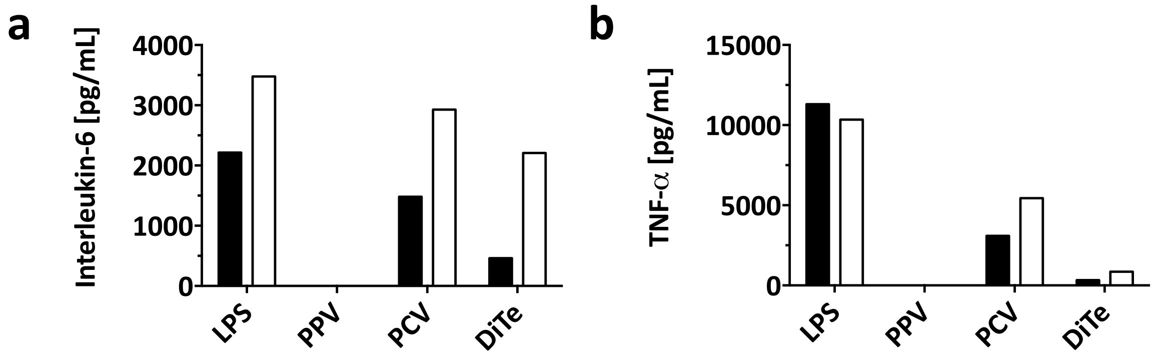

Supplementary Figure S3: Normal innate response. Strong local injection site reactions have been described in patients with over active innate immunity including Behcet's disease and STAT3 loss-of function mutations. To test this, 1 M PBMC were stimulated with 50 ng/mL lipopolysaccharide (LPS, TLR4 ligand) and cultured overnight. Culture supernatants were analysed by ELISA for Interleukin-6 (IL-6, Peprotech Switzerland IL-6-ELISA: 900-M16) (a) and TNFα (Peprotech Switzerland TNFa-ELISA: 900-TM25) (b). The patient (black bar) and control (open bar) showed comparable innate effector cytokine expression, with the control having higher IL-6 values.
